# Association of preoperative monocyte level with postoperative delirium in patients receiving cardiac surgery under propofol-based anesthesia: a retrospective study

**DOI:** 10.1186/s13019-026-04642-4

**Published:** 2026-07-20

**Authors:** Xuebin Wen, Jianbo Shan, Shuyu Deng, Gengfan Ye, Zhihao Pan

**Affiliations:** 1 Department of Anesthesiology, The Affiliated Lihuili Hospital of Ningbo University, Ningbo, 315040 Zhejiang China; 2 Department of Neurosurgery, The Affiliated Lihuili Hospital of Ningbo University, Ningbo, 315040 Zhejiang China

**Keywords:** Postoperative delirium, Cardiac surgical procedure, Observational study, MIMIC

## Abstract

**Background:**

Postoperative delirium is a common complication after cardiac surgery, and perioperative inflammation may contribute to its development. However, the association between preoperative blood indicators and postoperative delirium in patients receiving propofol-based anesthesia remains unclear.

**Objective:**

To identify preoperative blood indicators associated with postoperative delirium risk in patients undergoing cardiac surgery under propofol-based anesthesia.

**Methods:**

Related data of 694 patients were from MIMIC-IV database. Importance ranking using 2 machine learning algorithms was performed, followed by LASSO regression, multivariable logistic regression, and trend regression analyses to identify the important factors associated with postoperative delirium risk. Receiver operating characteristic curve and decision curve analysis were used to assess their clinical value for predicting delirium.

**Results:**

Among all patients, 44 patients experienced postoperative delirium (identified via ICD diagnostic coding during hospitalization). Machine learning algorithms initially implied the importance of monocyte in postoperative delirium risk as monocyte ranked first both in 2 algorithms. LASSO regression identified 7 variables, and 4 variables (red cell distribution width, red blood cell, monocyte, sepsis) showed independent association with delirium risk (all *P* < 0.05). Further, we found that the risk of delirium was increased with the increase of monocyte level (P for trend = 0.042), particularly when monocyte level exceeded 5.4% (*P* = 0.037). These results suggested monocyte as a key indicator independently associated with delirium risk and showing the most favorable discriminative performance among the evaluated markers, although its predictive utility requires prospective validation.

**Conclusions:**

Preoperative monocyte level was independently associated with coded postoperative delirium in this cohort, but prospective validation is still required before any predictive application.

## Introduction

Delirium has become a common postoperative complication, as the number of older patients undergoing major surgeries has increased. Delirium is mainly manifested as five key features: attention and consciousness disorder; these disorders occur in a short time and its severity fluctuates throughout the day; additional cognitive barriers; these disturbances cannot be better explained by other pre-existing neurocognitive disorders; and related evidence support that another medical condition causes the disturbance [1]. The potential mechanisms of postoperative delirium are unclear and likely to be multifactorial since no single factor can be used to clarify the phenomenon. The whole surgery-related delirium had a prevalence rate of 5%−50% [2]. Old patients had higher delirium risk, with an incidence rate of 30%−52% when age > 50 years and 33%−50% when age > 80 years [3, 4]. Due to the difference in diagnostic tools and study design, the incidence of postoperative delirium among patients undergoing cardiac surgery varies largely (11.4%−55%) [5]. Postoperative delirium can cause unfavorable outcomes including prolonged length of hospital stay, increased mortality, cognitive impairment, memory decline, and long-term care in cardiac surgery as well as other types of surgery [6]. It should be noted that postoperative delirium and postoperative cognitive decline share overlapping multifactorial mechanisms, and surgery-related inflammatory responses are considered one of the most plausible common pathways. Glumac et al. reported that preoperative corticosteroid administration attenuated the inflammatory response prompted by cardiac surgery and reduced early postoperative cognitive decline [7], further supporting the relevance of perioperative inflammation in postoperative neurocognitive disorders.

There are several risk factors associated with the delirium risk. Predisposing factors include smoking, hypertension, cardiac disease, sepsis, and premorbid dementia. Precipitating factors include respiratory failure and shock, metabolic disturbances, prolonged mechanical ventilation, and adverse environmental conditions [8]. The application of anesthetics is even more indispensable during surgical treatment, especially surgery is a major therapy for most of the cancer [9]. Anesthetics applications have been found to correlate with the cancer cell function and patient’s outcome [10, 11]. The use of anesthetics also influenced the outcome of non-cancer patients. The types of anesthetics were found to correlate with the postoperative delirium risk. Benzodiazepines, which are currently used less frequently in cardiac surgery, have been shown to be associated with the incidence of postoperative delirium in previous studies [12–14]. However, intraoperative midazolam administration during cardiac surgery may not be associated with postoperative delirium [15]. Although propofol shows a lower rate of postoperative delirium compared to sevoflurane, it has a higher rate when compared to dexmedetomidine sedation [16, 17]. In addition, the surgery type may influence the delirium risk associated with anesthetics types. It was found that old patients who received major cancer surgery had less delirium after propofol than sevoflurane anesthesia [18]. Dexmedetomidine caused a lower incidence of postoperative delirium than propofol in patients undergoing orthopedic surgery [19]. It followed that the effect of anesthetics on the postoperative delirium should not be ignored. However, anesthetics related delirium risk is inevitable to some extent.

Finding and managing risk factors are feasible and effective methods for reducing delirium risk. However, several risk factors, such as age, comorbidity, anesthetics application, and duration of mechanical ventilation, are non-modifiable factors. Therefore, focusing on these non-modifiable factors is of little value in preventing delirium risk. It is paramount to identify more modifiable factors (preoperative and intraoperative, not postoperative) in the prevention and management of postoperative delirium [20, 21]. For example, previous study explored related factors influencing postoperative delirium in elderly patients, finding that high preoperative plasma IL-6 levels (≥ 9 pg/mL) were significantly linked to the development of postoperative delirium [22]. Currently, there were several researches indeed focusing on the investigation of preoperative indicators. But few studies paid attention to the potential intraoperative factors. Although directly exploring intraoperative biological markers is an ideal approach to understanding the mechanism of postoperative delirium, due to the difficulty in obtaining them in real time in routine clinical practice, data in this field is extremely scarce at present, which constitutes a bottleneck in research. Therefore, under the current conditions, making full use of easily accessible preoperative indicators to maximize predictive and explanatory capabilities has become an urgent and practical alternative strategy.

In this study, we explored the association of several preoperative blood indicators with postoperative delirium. To control the potential influence of surgery type, anesthetics type, and anesthetize mode on the delirium risk, we only enrolled the patients who underwent cardiac surgery and received propofol anesthesia with the route of intravenous drip. This study aims to disclose related risk indicators that are inexpensive and easy to monitor during surgery treatment. We hypothesized that preoperative inflammatory-related blood indicators are independently associated with postoperative delirium in patients undergoing cardiac surgery under propofol-based anesthesia. Specifically, we posit that these real-time, peripheral biomarkers reflect the intensity of the surgical stress response, which may reflect perioperative inflammatory status associated with vulnerability to postoperative delirium rather than establish a direct mechanistic pathway.

## Methods

### Participants

This was a retrospective observational study based on the Medical Information Mart for Intensive Care (MIMIC)-IV database, a publicly available single-center critical care database containing data from Beth Israel Deaconess Medical Center, Boston, MA, USA, collected between 2008 and 2019. All patient-related information was anonymous and informed consent was not required. Patients who met the following criteria were included in the study: [1] aged ≥ 18 years old; [2] received cardiac surgery; [3] received propofol anesthesia with the route of intravenous drip in the perioperative period; [4] with postoperative delirium status ascertainable from ICD diagnosis records during the index hospitalization; [5] length of hospital stay (LOS) ≥ 2 days; [6] without death in the hospital. For patients with multiple admissions records, we only retained the record of the patient’s first admission. Exclusion criteria included [1] absence of important data; [2] loss to data of LOS; [3] other sedation drugs were used; [4] delirium history before admission. Patients receiving other sedative agents were excluded to minimize confounding by sedative-specific delirium risk, as different anesthetics and sedatives have been reported to carry different delirium risks; patients who died during hospitalization were excluded because delirium ascertainment requires sufficient postoperative observation time and because in-hospital death represents a competing outcome. We acknowledge that both exclusions may have preferentially removed more severely ill patients, so the findings should be interpreted as applying to propofol-treated cardiac surgical patients who survived to discharge rather than the broader cardiac surgical population.

According to the recorded procedure information, the included cardiac procedures were mainly valve procedures and coronary artery procedures, such as aortic valve replacement-related procedures and coronary artery bypass grafting. All included patients received propofol through intravenous administration according to the medication records. Medication records may not fully capture anesthetic depth, exact duration, or concomitant adjunct agents, and this should be considered when interpreting the anesthetic exposure classification. However, detailed information on cardiopulmonary bypass management and standardized anesthesia protocols was not available in MIMIC-IV and therefore could not be further analyzed. Postoperative delirium was identified using postoperative ICD diagnosis records during the index hospitalization. Because MIMIC-IV does not provide a uniform prospective delirium assessment schedule, postoperative delirium was not defined at a fixed postoperative assessment time point.

### Data collection

In this study, the baseline characteristics of patients included gender (male, female), age (years), marital status (single, married, divorced, widowed), body mass index (BMI) category (non-obese, obese), first ICU type, and ICU stay requirement (yes, no). The comorbidities of patients included sepsis, respiratory failure, heart failure, shock, diabetes, hypertension, and coronary heart disease (CHD). The laboratory indicators were also collected including red cell distribution width (RDW, %), uric acid (mg/dL), urea nitrogen (mg/dL), platelets (K/uL), anion gap (mEq/L), hemoglobin (g/dL), red blood cell (m/uL), monocyte (%), lymphocyte (%), and creatinine (mg/dL). Laboratory indicators were collected from tests performed within 24 h after hospital admission. In addition, the surgery type data was also collected (coronary artery surgery, valve surgery). Because only the surgery start time was available in the database and a reliable surgery end time was not available, intraoperative laboratory measurements could not be rigorously defined. Therefore, the present analysis focused on preoperative laboratory indicators. Specifically, laboratory indicators were collected from tests performed within 24 h after hospital admission, and the sampling time of laboratory indicators was compared with the recorded surgery start time, and laboratory indicators measurements obtained before the start of surgery were defined as preoperative levels. We also collected the hospital-LOS (days) of patients. The number of patients with smoking and drinking history was too small, therefore, the 2 variables were removed from our final analysis.

### Statistical analysis

The categorical variables were expressed as frequency (%), and their distribution differences between delirium and non-delirium were analyzed by χ^2^ test. Continuous variables with normal distribution were expressed as mean and standard deviation, and differences between the 2 groups were analyzed by t-test. The data with abnormal distribution was expressed as median and quartile, and differences between the 2 groups were analyzed by the Mann-Whitney U test. The variables showing differences between the 2 groups were firstly evaluated the importance degree by 2 machine learning algorithms including Random forest and XGBoost. Next, these variables were enrolled to Least absolute shrinkage and selection operator (LASSO) regression analysis. This method is a compressed estimation. It gets a more refined model by introducing a penalty coefficient (λ), which compress some coefficients and set some coefficients to zero. When the optimal LASSO model was achieved, the retained variables with non-zero coefficients were the key factors. Multivariable logistic regression analysis using a Enter method was conducted to explore the independent association of these key factors with postoperative delirium risk. Because the candidate variables had already undergone two rounds of pre-selection (importance ranking and LASSO), the Enter method was deliberately used so that all 7 pre-selected variables entered the model simultaneously as a single confirmatory step rather than as a further exploratory selection stage. Collinearity among these variables was assessed using variance inflation factors, all of which were below 1.5, indicating no meaningful multicollinearity. In addition, Receiver Operating Characteristic (ROC) analysis and Decision Curve Analysis (DCA) were used to assess the performance and obtained clinical net benefit of key factors for predicting the postoperative delirium. Trend regression analysis was performed to explore the influence of key factors changes on the postoperative delirium risk. Interaction effect analysis was conducted to explore the potential factors that influenced the association stability. All the analysis was performed by SPSS and R software. The optimal cutoff values used for sensitivity, specificity, and Youden index calculations were derived post hoc from the ROC curve within the same dataset rather than pre-specified, and no additional internal validation was performed for these ROC estimates beyond the 5-fold cross-validation used for LASSO tuning parameter selection; these ROC-based metrics should therefore be interpreted as descriptive of this cohort rather than externally validated. Because delirium incidence was low (6.3%), raw classification accuracy is not reported as a primary metric, as it can be dominated by the majority (non-delirium) class and does not reliably reflect discriminative performance; AUC, sensitivity, specificity, and Youden index are used instead to characterize discriminative performance. Two-sided *P* < 0.05 was considered statistically significant. In this study, a priori sample size calculation was not performed because this was a retrospective study based on a public database, and the final sample size was not prospectively determined by the investigators but determined by the predefined eligibility criteria and data availability.

## Results

### Baseline characteristics

In this study, a total of 694 patients treated with propofol were enrolled. Of whom, 44 patients experienced postoperative delirium. The baseline characteristics of patients undergoing cardiac surgery were presented in Table 1. There were differences between non-delirium and delirium groups in terms of ICU stay requirement, sepsis, heart failure, diabetes, RDW level, hemoglobin, red blood cell, monocyte, lymphocyte, and creatinine (all *P* < 0.05). The patients with a history of sepsis, heart failure, and diabetes had a larger proportion of delirium patients. However, patients requiring the ICU stay had a lower proportion of delirium. In addition, patients with delirium expectedly had longer length of hospital stay (*P* < 0.001). These differential variables were enrolled to the subsequent analyses.

**Table 1:** Baseline characteristics of patients undergoing cardiac surgery

	Subgroups	Non-delirium (*n* = 650)	Delirium (*n* = 44)	*P*
Gender	male	445 (93.9)	29 (6.1)	0.725
	female	205 (93.2)	15 (6.8)	
First ICU type	CVICU	379 (96.9)	12 (3.1)	0.360
	CCU	89 (93.7)	6 (6.3)	
	MICU+SICU	24 (92.3)	2 (7.7)	
	TSICU+Neuro SICU	5 (100.0)	0 (0.0)	
Obese	no	448 (92.8)	35 (7.2)	0.233
	yes	148 (95.5)	7 (4.5)	
ICU stay need	no	153 (86.4)	24 (13.6)	< 0.001
	yes	497 (96.1)	20 (3.9)	
Marital status	single	133 (91.7)	12 (8.3)	0.770
	married	387 (93.5)	27 (6.5)	
	divorced	47 (94.0)	3 (6.0)	
	widowed	46 (95.8)	2 (4.2)	
Sepsis	no	626 (94.6)	36 (5.4)	< 0.001
	yes	24 (75.0)	8 (25.0)	
Respiratory failure	no	541 (94.4)	32 (5.6)	0.076
	yes	109 (90.1)	12 (9.9)	
Heart failure	no	314 (97.5)	8 (2.5)	< 0.001
	yes	33 6 (90.3)	36 (9.7)	
Shock	no	549 (94.2)	34 (5.8)	0.208
	yes	101 (91.0)	10 (9.0)	
Diabetes	no	407 (96.0)	17 (4.0)	0.002
	yes	243 (90.0)	27 (10.0)	
Hypertension	no	230 (93.5)	16 (6.5)	0.895
	yes	420 (93.8)	28 (6.3)	
CHD	no	208 (96.3)	8 (3.7)	0.055
	yes	442 (92.5)	36 (7.5)	
Surgery Type	Coronary Artery Surgery	218 (95.2)	11 (4.8)	0.244
	Valve Surgery	432 (92.9)	33 (7.1)	
Age		65 [56, 73]	68 [60, 76]	0.153
RDW (%)		13.6 [12.8, 14.8]	15.5 [13.6,16.5]	< 0.001
Uric acid (mg/dL)		7.1 [5.6, 9.4]	12.2 [6.8, 12.2]	0.766
Urea nitrogen (mg/dL)		491 [250, 661]	353 [318, 471]	0.453
Platelets (K/uL)		120 [90, 160]	147 [90, 181]	0.094
Anion gap (mEq/L)		10 [9, 12]	10 [9, 12]	0.375
Hemoglobin (g/dL)		8.6 [7.5, 10.2]	7.6 [6.9, 8.5]	< 0.001
Red blood cell (m/uL)		2.93 [2.52, 3.41]	2.49 [2.36, 3.03]	< 0.001
Monocyte (%)		4.1 [3.0, 5.9]	7.2 [4.5, 8.9]	< 0.001
Lymphocyte (%)		14.1 [8.3, 20.7]	8.9 [5.5, 13.5]	0.002
Creatinine (mg/dL)		0.8 [0.7, 1.1]	1.1 [0.9, 1.8]	< 0.001
Hospital length of stay (days)		7.9 [5.4, 12.1]	13.2 [7.7, 26.1]	< 0.001

BMI, body mass index; CHD, coronary heart disease; CVICU, Cardiac Vascular Intensive Care Unit; CCU, Coronary Care Unit; MICU/SICU, Medical/Surgical Intensive Care Unit; TSICU, Trauma SICU; RDW, red cell distribution width. Obese was defined as BMI≥28.0 kg/m^2^

### Key factors identification of postoperative delirium

**Fig. 1:**
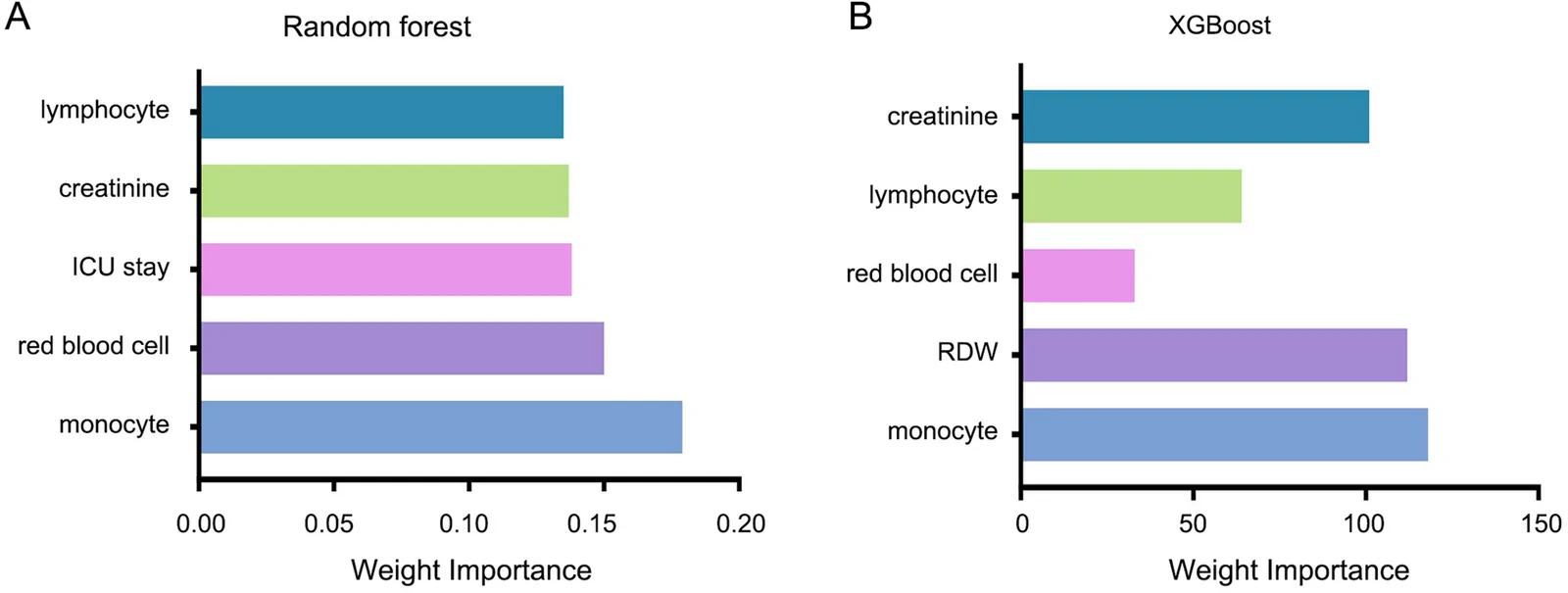
The importance evaluation of differential variables on the postoperative delirium risk. (**A**) Two machine learning algorithms were used to rank the importance of variables showing difference between non-delirium and delirium groups using (**A**) Random forest and (**B**) XGBoost. The top 5 variables in each algorithm were presented

Regarding these differential variables, 2 machine learning algorithms were initially used to assess their importance on the postoperative delirium risk. Two algorithms determined 4 common factors containing red blood cell, monocyte, lymphocyte, and creatinine, and all indicated that the importance of monocyte ranked first (Fig. 1A and B). Importance ranking analysis initially highlighted the importance of monocyte on the postoperative delirium risk.

**Fig. 2:**
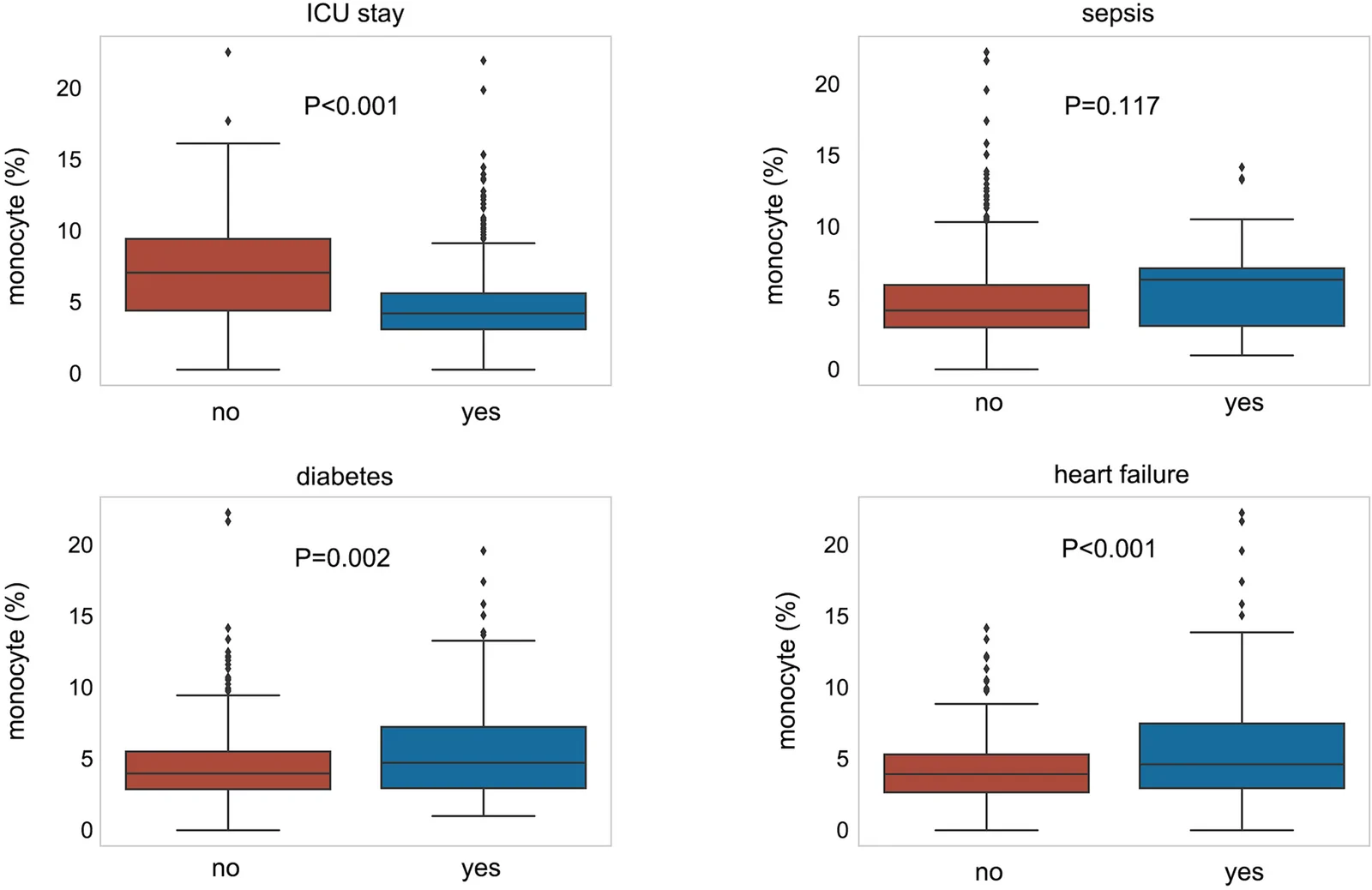
The comparison of monocyte level among different subgroups stratified by ICU stay requirement, sepsis, diabetes, and heart failure

We next compared the monocyte level among different subgroups (Fig. 2), finding that monocyte level was higher in patients with heart failure (*P* < 0.001) and diabetes (*P* = 0.002). However, the patients without ICU stay requirement had higher monocyte level (*P* < 0.001). Monocyte level showed no difference between patients with or without sepsis.

**Fig. 3:**
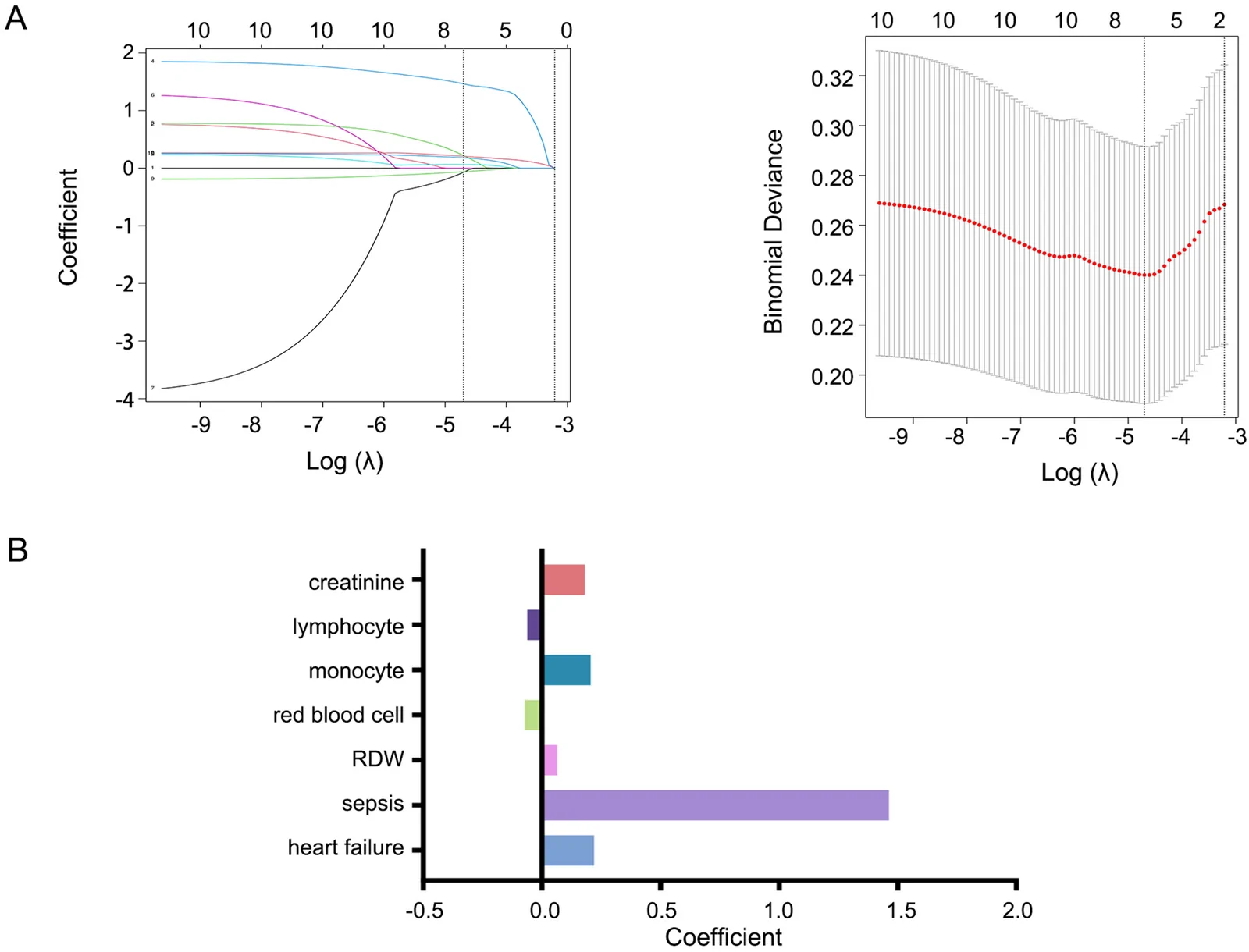
The key factors identification associated with postoperative delirium risk by LASSO (least absolute shrinkage and selection operator) regression analysis. (**A**) LASSO coefficient profiles. (**B**) Five time cross-validation for tuning parameter selection in the LASSO model. (**C**) The coefficients of key factors within the optimal LASSO model

Besides the importance ranking, we also performed LASSO and logistic regression analyses to identify the key factors associated with delirium risk. The variables showing differences between non-delirium and delirium groups were enrolled to the LASSO regression analysis to filter the redundant variables. When the minimum mean square error was set as 0.009, the optimal LASSO model was achieved which including 7 non-zero variables, namely RDW, red blood cell, monocyte, lymphocyte, creatinine, heart failure, and sepsis (Fig. 3A). Their LASSO coefficients were presented in Fig. 3B.

Next, the role of 7 variables within optimal LASSO model was evaluated by multivariable logistic regression analysis to explore their independent association with postoperative delirium risk (Table 2). Complete data for all 7 variables were available in 495 patients, including 31 delirium events (approximately 4.4 events per variable). The results showed that red blood cell (OR = 0.373, *P* = 0.012), monocyte (OR = 1.148, *P* = 0.007), RDW (OR = 1.348, *P* = 0.008), and sepsis (OR = 4.317, *P* = 0.012) were independently related to delirium risk. Other variables showed no independent association with the postoperative delirium. It followed that there were 2 variables showing the independent role in the postoperative delirium risk among the common 4 variables in importance ranking analysis, namely red blood cell and monocyte.

**Table 2:** The association between variables within optimal LASSO model and postoperative delirium risk by multivariable logistic regression analysis

Variables	Estimate	OR	95%CI	*P*
(Intercept)	−5.589	0.004	[0.000, 0.131]	0.002
RDW	0.299	1.348	[1.082, 1.687]	0.008
Red blood cell	−0.985	0.373	[0.166, 0.779]	0.012
Monocyte	0.138	1.148	[1.038, 1.271]	0.007
Lymphocyte	−0.035	0.966	[0.905, 1.022]	0.265
Creatinine	0.221	1.247	[0.916, 1.617]	0.106
Heart failure	0.550	1.733	[0.645, 5.182]	0.293
Sepsis	1.463	4.317	[1.315, 13.259]	0.012

OR, odds ratio; CI, confidence interval

### Clinical value of independent factors for predicting the postoperative delirium

**Fig. 4:**
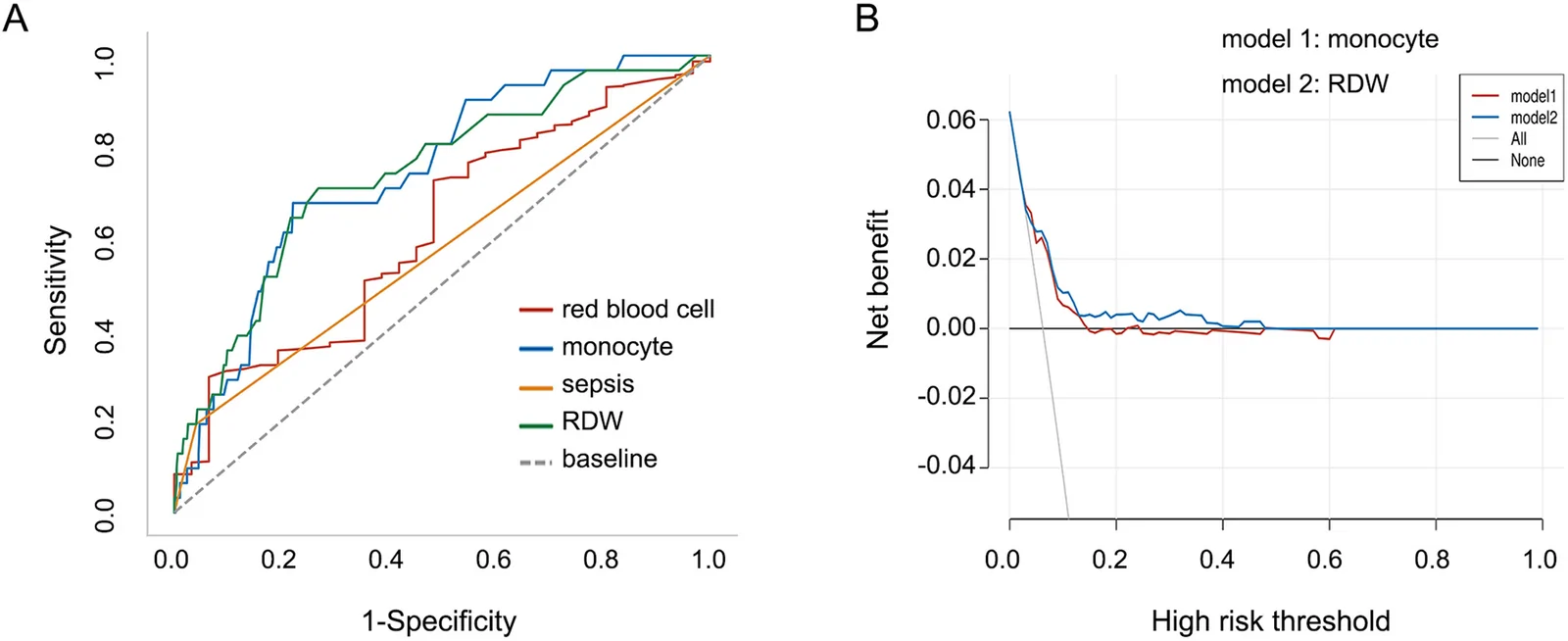
Clinical value evaluation of key factors for predicting the postoperative delirium. (**A**) ROC (receiver operating characteristic) analysis. (**B**) DCA (decision curve analysis) analysis was used to identify the clinical net benefit of monocyte and RDW (red cell distribution width)

The performance of the 4 independent variables for predicting the postoperative delirium was then assessed by ROC analysis (Table 3; Fig. 4A). The results showed that red blood cell had highest sensitivity and sepsis had highest specificity. The AUC values of monocyte, red blood cell, RDW, and sepsis for predicting postoperative delirium were 0.744, 0.620, 0.742, and 0.576, respectively. Monocyte showed the highest AUC value (0.744), Youden index (0.456). Delong test indicated that the AUC of monocyte was significantly higher than that of red blood cell and sepsis (all *P* < 0.05). These findings suggest that monocyte was the blood indicator most strongly associated with coded postoperative delirium in this cohort and showed relatively favorable discriminative performance among the evaluated markers. ROC analysis showed that the best cutoff value of monocyte was 6.3.

**Table 3:** The clinical value of independent factors for predicting postoperative delirium by ROC

Variables	Monocyte	Red blood cell	Sepsis	RDW
AUC	0.744	0.620	0.576	0.742
Sensitivity	0.677	0.727	0.194	0.71
Specificity	0.778	0.516	0.959	0.731
Youden index	0.456	0.243	0.153	0.441
Best cutoff	6.3	2.5	1	14.6
Delong test	/	< 0.001	0.003	0.960

Accuracy is not presented because, at a delirium incidence of 6.3%, this metric is strongly influenced by the prevalence of the majority class and does not reliably reflect discriminative performance (e.g., a classifier predicting no delirium for all patients would achieve ~ 93.9% accuracy without any true discriminative ability); AUC, sensitivity, specificity, and Youden index are reported instead. Abbreviations: AUC, area under the curve; RDW, red cell distribution width

It should be noted that the AUC of RDW ranked second place. Therefore, we also compared the clinical net benefits of monocyte and RDW by DCA method (Fig. 4B), finding that the obtained net benefit of RDW for predicting postoperative delirium was more favorable than monocyte, although monocyte had higher AUC than RDW. These results also implied the importance of RDW for predicting postoperative delirium.

### The association and stability of monocyte with postoperative delirium

Further, we performed a trend regression analysis to explore the influence of monocyte changes on the postoperative delirium risk by setting monocyte level as a categorical variable according to the tertile of monocyte (Table 4). The results showed that with the increase of monocyte level, the risk of postoperative delirium increased (adjusted P for trend = 0.042). Especially, when the level of monocyte was greater than 5.4, the postoperative delirium of patients was significantly increased (adjusted *P* = 0.037).

**Table 4:** The association between monocyte and postoperative delirium by trend regression

Variables	Crude model		Adjusted model	
	OR (95%CI)	*P*	OR (95%CI)	*P*
Q1 (0.0, 3.5	Reference		Reference	
Q2 (3.6, 5.3)	4.447 [0.930, 21.268]	0.062	4.566 [0.910, 22.897]	0.065
Q3 (5.4, 22.6)	11.990 [2.765, 51.995]	0.001	5.098 [1.100, 23.630]	0.037
P for trend		< 0.001		0.042

OR, odds ratio; CI, confidence interval

To assess the association stability of monocyte with postoperative delirium, we performed an interaction effect analysis to explore the potential influence of other variables on their association. We considered 3 important comorbidities including sepsis, heart failure, and diabetes (as they showed differences between delirium and non-delirium groups in baseline analysis). In addition, age and gender were the 2 common features generally associated with several human disease, we also considered them although they showed no statistical difference in baseline analysis. The result of interaction effect analysis was presented in Table 5, finding that these 5 features all showed no interaction effect with monocyte (all *P* > 0.05). Interaction effect analysis indicated the stable association between monocyte and postoperative delirium. As monocyte percentage may be influenced by fluctuations in other leukocyte subsets, we additionally conducted the supplementary analyses on absolute monocyte count. The results showed that absolute monocyte count remained significantly associated with postoperative delirium (OR = 2.410, *P* = 0.008) and showed discriminative performance (AUC = 0.692) comparable to monocyte percentage in the same subset (AUC = 0.743), supporting the robustness of monocyte as a delirium-associated indicator regardless of the metric used.

**Table 5:** The interaction effect analysis between key features and monocyte

	Estimate	OR	95%CI	*P* for interaction effect
Age* monocyte	−0.004	0.996	[0.988, 1.004]	0.347
Gender* monocyte	0.121	1.128	[0.940, 1.385]	0.216
Sepsis* monocyte	−0.049	0.952	[0.732, 1.259]	0.716
Heart failure* monocyte	−0.182	0.833	[0.644, 1.092]	0.164
Diabetes* monocyte	−0.118	0.888	[0.738, 1.060]	0.193

*The Symbol denotes an interaction term between the two variables

OR, odds ratio; CI, confidence interval

## Discussion

In this study, we found that preoperative monocyte level was independently associated with postoperative delirium and showed relatively favorable discriminative performance among the evaluated blood indicators in patients undergoing cardiac surgery under propofol-based anesthesia. Delirium is a common postoperative brain dysfunction that can increase the risk of long-term severe cognitive impairment and mortality [23]. This study aimed to identify significant modifiable preoperative factors correlated with postoperative delirium. Our results initially revealed that the prevalence of postoperative delirium after cardiac surgery was 6.34%, which is lower than the 13.0%−16.1% reported in previous studies using the MIMIC database [24–26]. This discrepancy may be attributed to variations in anesthetics, cardiac surgery types, and patient characteristics. Notably, postoperative delirium in this study was ascertained exclusively through ICD diagnostic coding during hospitalization rather than through validated bedside instruments such as the Confusion Assessment Method for the ICU (CAM-ICU) or DSM-based assessment. Administrative coding is known to substantially underestimate true delirium incidence, particularly for hypoactive presentations; in a validation study against clinical assessment, the sensitivity of ICD-coded delirium was only 46.3% [27]. The 6.34% incidence observed here should therefore be regarded as a coding-based lower-bound estimate rather than the true clinical incidence, and coding practices, ICU documentation intensity, and illness severity may have influenced case ascertainment as much as true biological risk. This limitation is consistent with the counterintuitive finding that patients not requiring ICU stay had a lower proportion of coded delirium (Table 1) despite a numerically higher comorbidity burden (median Charlson comorbidity index 7 vs. 5 for patients requiring ICU stay); this pattern is more consistent with a shorter, less intensively monitored hospitalization (median length of stay 5.9 vs. 8.6 days) reducing the opportunity for delirium to be observed and coded, rather than a true protective effect of avoiding ICU care. Given these ascertainment limitations, the associations reported should be interpreted as relating to clinically coded delirium. The anaesthetic used was limited to propofol in this study, which has been shown to result in a lower incidence of postoperative delirium compared to sevoflurane (8.8% vs. 34.2%) [18]. The relatively low coded incidence may also reflect the restrictive eligibility criteria and the focus on a specific propofol-treated cohort.

Anesthetics is essential in surgery. Previous researches have indicated that local anesthetics can influence cancer prognosis in cancer surgery [28–30]. The potential influence of anesthetics in non-cancer patients is also noteworthy. In cardiac surgery, different anesthetics may lead to varying postoperative cognitive function changes [31, 32], as intravenous anesthesia based-propofol resulted in a higher incidence of postoperative cognitive deficits compared to sevoflurane or desflurane. The study also highlighted the importance of anesthesia mode on postoperative cognitive function, which find that intravenous anesthesia based on propofol has demonstrated better brain protection with a low incidence of cognitive dysfunction compared to inhalational anaesthesia [33, 34]. The influence of anesthetics on the cognitive dysfunction has been widely revealed. In this study, all the patients received the intravenous drip of propofol. The mechanism behind propofol-induced delirium may be related to its interaction with muscarinic acetylcholine receptors [35]. Intravenous anesthetics may suppress normal immune responses, potentially exacerbating already compromised defenses [36]. In contrast to inhalational anesthetics such as isoflurane, propofol maintains the bactericidal function of alveolar macrophages during anesthesia [37]. Propofol did not inhibit T-lymphocyte proliferation or leukocyte function compared to sodium thiopental and etomidate [38–40]. In addition, propofol can induce a higher postoperative fraction of the CD163 (high) HLA-DR (low) monocyte subset, which represented either a compensatory mechanism dampening the higher inflammatory condition [41]. Propofol also can allow mononuclear cells to retain their cytotoxicity by protecting immune cells from apoptosis [36]. These findings revealed the potential regulation of propofol on immune microenvironment. Whereas propofol seemed to induce mixed pro- and anti-inflammatory effects on the immune system [42]. The alteration of immune microenvironment can corresponding induce the changes of inflammatory factors. Studies on healthy volunteers suggest that propofol might inhibit IL-6 and IL-10 production by lipopolysaccharide-stimulated monocytes in vitro [43]. However, in patients undergoing cancer surgery, higher postoperative levels of anti-inflammatory cytokines (IL-10) were observed with intravenous anesthesia propofol compared to inhalational anesthesia [30]. These findings highlighted the importance and dural role of propofol on the immune responses and inflammation.

Increasing research evidence indicates that neuroinflammation may contribute to postoperative delirium progression [44, 45]. Neuroinflammation is a known pathological hallmark of neurodegenerative disorders including delirium [46], and it is central to the pathology of brain diseases. In this study, we identified three preoperative indices associated with postoperative delirium in patients received cardiac surgery under propofol-based anesthesia: RDW, red blood cell, and monocytes. Among the evaluated markers, monocyte showed relatively favorable discriminative performance for coded postoperative delirium and was therefore examined in greater detail. RDW has been reported as an important marker of neuroinflammation [47]. Red blood cell function disturbances play a vital role in neurodegenerative disease pathophysiology [48]. Monocytes, a subset of white blood cells, account for 3% to 10% of total leukocytes in adults and play a crucial role in immune and inflammatory regulatory pathways [49]. According to the Warburg effect, changes in metabolic pathways within monocytes can lead to inflammatory stimuli in cardiac surgery and extracorporeal circulation [50]. Plasma cytokine levels of the inflammatory cytokines IL-6 and IL-8 are increased in patients undergoing cardiac surgery [51–53]. It has been suggested that monocyte mobilization and stimulation may enhance inflammatory responses and increase pro-inflammatory factor and chemokine expression, which may potentially contribute to neuroinflammation [54]. 

In this study, we found that monocytes were significantly and independently associated with the risk of postoperative delirium in patients received cardiac surgery under propofol-based anesthesia by multiple statistical methods. The study also found that higher preoperative monocyte levels increased the risk of delirium [55]. In addition, previous studies indicated the relationships between cardiac surgery and monocyte level. Fingerle-Rowson et al. found an increase in intermediate monocytes in patients who had previously undergone cardiac surgery [56]. Mossanen’s prospective observational study noted that post-cardiac surgery, delirium patients experienced a reduction in monocytes immediately after surgery, followed by a significant increase on days 1 and 4, particularly in classical and intermediate monocytes [57]. The study also reported that surgery and general anesthesia with intravenous propofol, during and in the short term after the procedure, induced a neuroinflammatory response dominating by monocyte attractants [58]. These acknowledges indicated the monocytes levels can be regulated by several factors such as surgery and anesthetics.

We further explored the potential mechanisms of delirium associated with monocytes. The mechanistic discussion below draws on prior experimental and clinical literature to generate biological hypotheses; because our retrospective design lacked perioperative cytokine measurements, neuroinflammatory biomarkers, or longitudinal immune profiling, it does not by itself demonstrate that monocytes mechanistically mediate neuroinflammation leading to delirium in our cohort, and should be interpreted as hypothesis-generating rather than confirmatory. An increase in monocytes indicates a higher level of infection, signifying an ongoing harmful inflammatory response. During such a response, it is necessary to simultaneously activate counter-regulatory anti-inflammatory mechanisms to offset potential harmful effects. The key components of this anti-inflammatory response are anti-inflammatory cytokines (e.g., IL-10), stress hormones, and neuroinhibitory responses [59]. In addition, the afferent neural pathways of the nervous system response are peripheral nerves that detect and transmit signals about infection or injury to the central nervous system. These signals then trigger the CNS’s efferent pathways via the release of acetylcholine through the vagus nerve. This acetylcholine release dampens the production of tumor necrosis factor, interleukin-6, and interleukin-1β by immune cells expressing the NACH α−7 subunit. Consequently, this may contribute to dysregulated neuroimmune responses that have been hypothesized to participate in delirium pathophysiology [60, 61]. Several neuromodulation therapies have been reported to attenuate neuroinflammation such as transcranial magnetic stimulation, transcranial electrical stimulation, deep brain stimulation, and vagus nerve stimulation [62]. Providing neuromodulation therapy before operation seems to be a promising choice for inhibiting neuroinflammation and postoperative cognitive dysfunction.

Finally, several limitations should be stated. In this study, we only focused on postoperative delirium, the data on postoperative cognitive dysfunction were not obtained. Moreover, the patients in this study all received propofol anesthesia. We failed to evaluate the difference in postoperative delirium risk due to the anesthetic type as the sample size of patients who received other anesthetics was small. In addition, different cardiac surgical procedures may carry different risks of postoperative delirium, but the limited positive events did not allow more detailed procedure-stratified analyses. Detailed cardiopulmonary bypass and anesthesia management data were also unavailable in MIMIC-IV. Moreover, postoperative delirium was identified from in-hospital diagnostic coding rather than from a standardized serial bedside assessment at fixed postoperative time points [27]. The multivariable model included 7 variables but only 31 delirium events among patients with complete data (*n* = 495), yielding an events-per-variable ratio of approximately 4.4, below the conventional threshold of 10 [63]; although collinearity was not evident (all VIF < 1.5), this low ratio may have led to unstable effect estimates, consistent with the wide confidence intervals observed for some variables (e.g., sepsis, heart failure), and these regression results should be interpreted cautiously. Additionally, patients who received other sedative agents or who died during hospitalization were excluded by design; while intended to isolate a homogeneous propofol-exposed, surviving cohort, these exclusions may have preferentially removed more severely ill patients, potentially limiting generalizability to the broader cardiac surgical population.

## Conclusions

This study suggests that a higher preoperative monocyte level was independently associated with coded postoperative delirium in this cohort and showed relatively favorable discriminative performance among the evaluated blood indicators; however, its predictive utility requires prospective validation.

## Data Availability

The datasets generated during and/or analyzed during the current study are available from the corresponding author on reasonable request.
